# The State Insane Hospital

**Published:** 1869-12

**Authors:** 


### Editorial.

### *The State Insane Hospital.*

DR. McFARLAND, Superintendent of the Insane Hospital, has published the following card:

NOTICE TO THE PUBLIC.—Against the better judgment of the undersigned, the practice has hitherto obtained of admitting general visitors to the apartments of the insane. It has been allowed under the idea that it might serve to allay the distrust existing in some minds toward all such institutions, and perhaps it may, to some extent, have had this effect. The existence of two boards of supervision—the board of trustees, and the board of state charities, whose examinations are frequent and thorough—leaves no longer a protest for a usage at once indelicate and inhuman, considering the character and object of the major class of visitors. The public are therefore respectfully informed that under a sense of duty to the afflicted, this practice will henceforth be discontinued, and residents of the vicinity of this institution will confer a favor, by stating to friends coming from abroad the reason why a practice, laudable as to the other state institutions in Jacksonville, must find its exception here.

This is an eminently proper measure, and we trust will be rigidly enforced. Every instinct of humanity revolts from the idea of having one's friends, thus fearfully diseased, exposed to the gaze of the idly curious, like wild animals in a menagerie. In the large majority of instances, such publicity seriously injures the patient, and either retards or imperils recovery. Every proper safeguard is thrown around the unfortunate inmates, so that none can be wrongly treated or restrained. Friends can always be reasonably indulged, but the mere sight-seer should be rigorously excluded.

Those who know the superintendent, Dr. McFarland, personally, need no guarantee that whatever kindness, zealous care, and professional skill can accomplish for his patients, will most certainly be secured.

Insanity is the result of physical disease of such a character that physiological rest of the affected organ is imperatively demanded. How can this be secured when the poor patient has, every day, to be "interviewed" by some sensational reporter?

### *Kansas City College of Physicians and Surgeons.*

THIS is the title of a new candidate for Western professional favor. The first, a preliminary and partial course, commenced on the first Monday of the present month. The second, which is to be a full course, is announced to commence the first Monday of October, 1870. Simeon S. Todd, M.D., is Dean of the Faculty, and if his colleagues equal him in energy and ability, an excellent institution will be built up on the far-away banks of the Missouri. We trust the new school may both deserve and achieve success.

### *Credit Omitted.*

DR. EDWARDS' case of Ovariotomy, under the department of *Loot*, in the last number, accidentally was not credited to our valued contemporary, the *Leavenworth Medical Journal*. Its location, however, showed very plainly that there was no intentional "trenching on our neighbor's preserves."

### *New Medical Journal.*

FROM distant Oregon comes the first number of a sprightly and neatly-clad *Medical and Surgical Reporter*. It is edited by E. R. Fiske, A.M., M.D., one of the Faculty of Willamette University, assisted by an able corps of collaborators. $4.00 *per annum*, in advance. We have marked passages for selection. Meanwhile, success to the *Oregon Reporter!* Address as above, Salem, Oregon.

THE *Phrenological Journal* has now reached its *fiftieth* volume, and celebrates its birthday by changing from the quarto to the more convenient octavo form. Without indorsing its peculiar views of *chart* phrenology, we may say that it contains a large amount of entertaining and instructive matter, and is afforded at $3.00 a year. Specimen copies, 15 cents. S. R. Wells, publisher, 389 Broadway, New York.

